# Eruptive xanthomas: a case report with literature review focusing on pathogenesis and clinical management

**DOI:** 10.1515/biol-2025-1327

**Published:** 2026-05-25

**Authors:** Xianhua Qiao, Xinglong Yu, Juanjuan Gao, Linfang Yang, Fang Cheng

**Affiliations:** Xingtai People’s Hospital, Xingtai, Hebei, 054035, China; Hebei Vocational University of Technology and Engineering, Xingtai, Hebei, 054000, China

**Keywords:** eruptive xanthomas, hypertriglyceridemia, case report, dyslipidemia, type 2 diabetes mellitus

## Abstract

A typical case of eruptive xanthomas secondary to untreated type 2 diabetes mellitus complicated with severe hyperlipidemia was reported. The patient had a history of acute pancreatitis one month before the onset of xanthomas. Laboratory tests revealed high levels of triglycerides, cholesterol, blood glucose, and acute inflammatory markers. Histopathological examination showed the foam cells in the dermis. After interventions, including a low-fat and low-carbohydrate diet, anti-diabetic therapy and lipid-lowering therapy, the patient’s skin lesions partially resolved within two months. At the same time, all laboratory indicators improved. In addition, a comprehensive literature review was conducted on the pathophysiological mechanisms, key clinical diagnostic points, triglyceride level thresholds, and main treatment strategies of eruptive xanthomas. This study emphasizes that eruptive xanthomas and acute pancreatitis are clinical manifestations induced by severe hypertriglyceridemia. For dermatologists, the occurrence of eruptive xanthomas should be used as a trigger to screen for dyslipidemia to effectively prevent life-threatening severe complications.

## 1 Introduction

The skin is a mirror that reflects the internal health status of the body. Many initial or accompanying symptoms of systemic diseases often manifest on the skin. Eruptive Xanthomas (EX) is a typical example of a reactive skin disorder characterized by a sudden appearance of yellow-red papules [1]. Although skin lesions are benign, they are almost always cutaneous markers of severe hypertriglyceridemia [2]. Severe hypertriglyceridemia, especially when the serum triglyceride level exceeds 1,000 mg/dL (about 11.3 mmol/L), will significantly increase the risk of acute pancreatitis. It is a severe hypertriglyceridemia with high morbidity and mortality [3], 4]. Long-term hypertriglyceridemia is an independent risk factor for atherosclerotic cardiovascular disease (ASCVD) [5], 6].

The development of eruptive xanthomas is closely related to severe lipid metabolism disorders. It is commonly seen in hereditary hyperlipoproteinemia or secondary to other conditions. The most common are uncontrolled diabetes, obesity, excessive alcohol consumption, and the use of certain medications [2]. Accurate identification and timely diagnosis of eruptive xanthomas are therefore crucial for uncovering potential life-threatening metabolic disorders. This study presents a typical case of eruptive xanthomas associated with uncontrolled type 2 diabetes, and conducts in-depth discussions based on recent research literatures. Its aim is to enhance clinicians’ understanding of this disease and standardize its diagnostic and therapeutic process.

## 2 Case presentation

### 2.1 Patient demographics and clinical history

**Figure 1:**
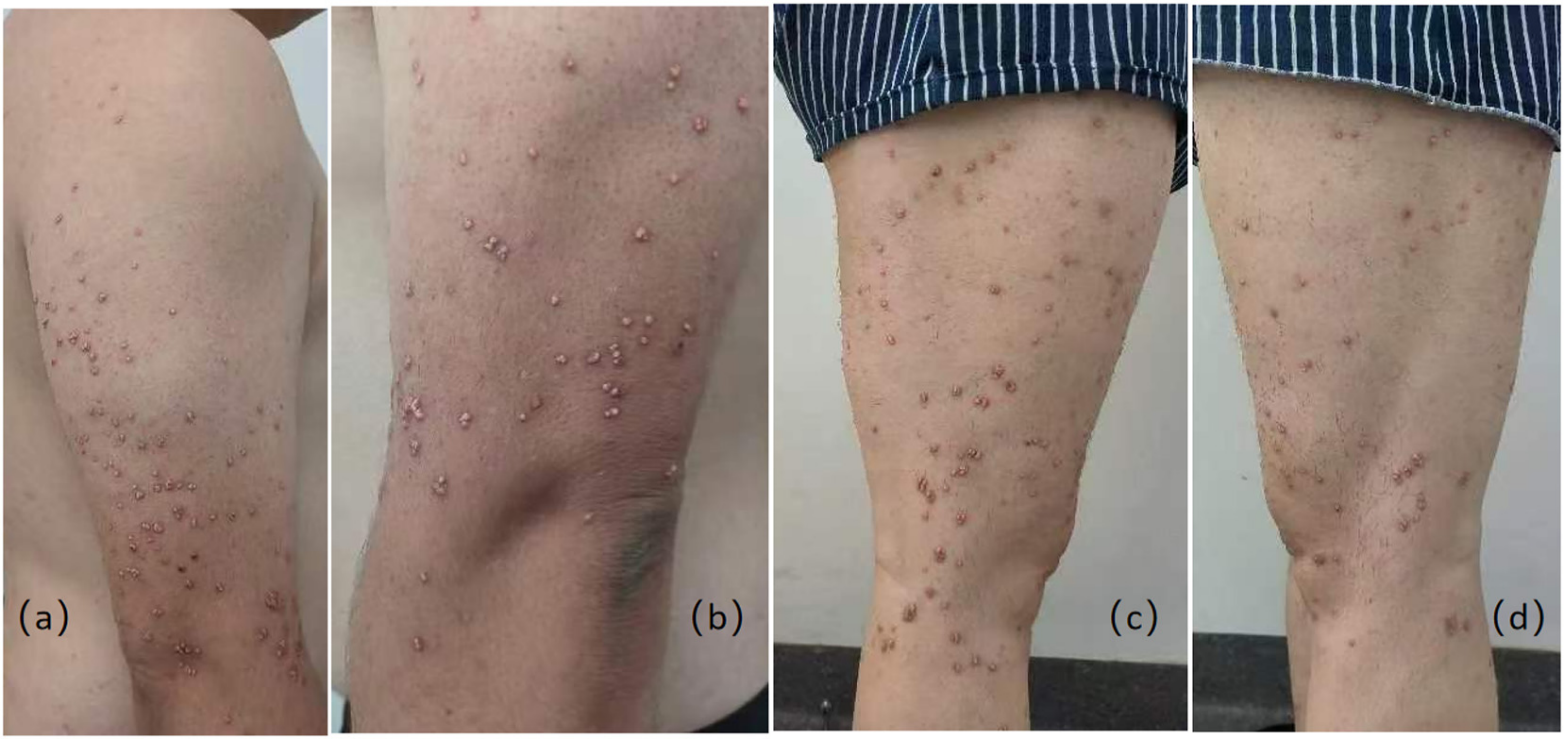
The area of the patient’s eruptive skin rash ((a): Right arm; (b): Left arm; (c): Right leg; (d): Left leg).

**Figure 2:**
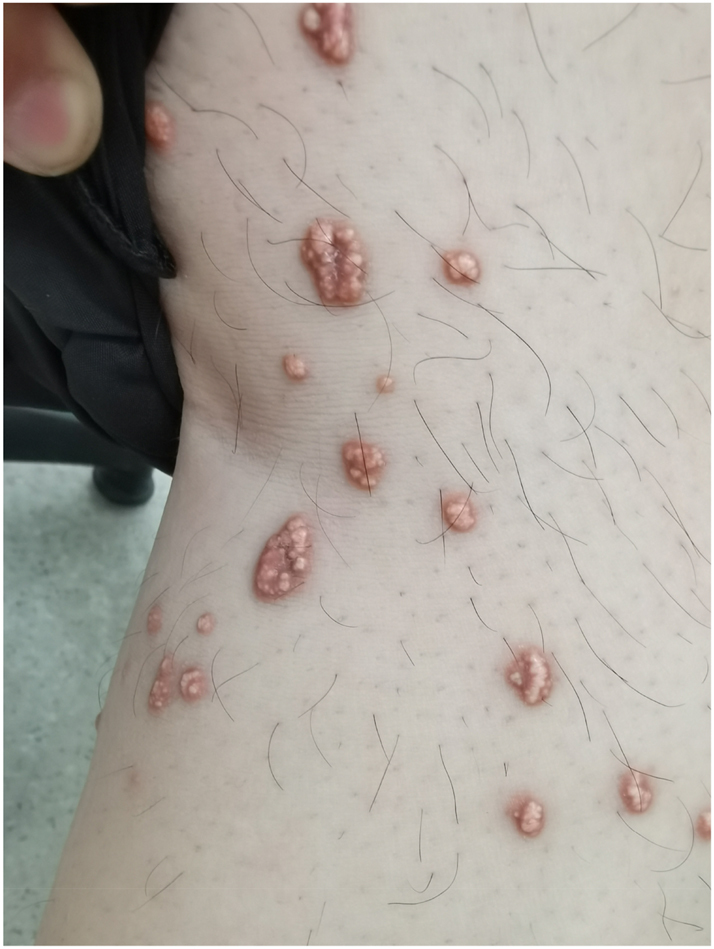
The area of the largest eruptive skin rash on the patient’s right leg.

**Figure 3:**
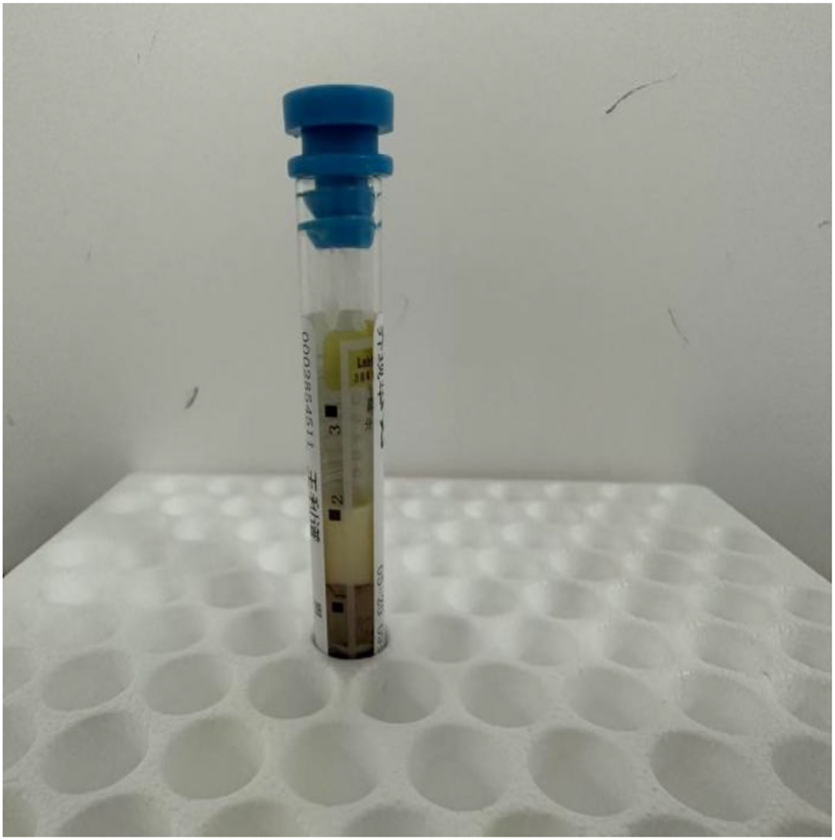
Chylous appearance of the fasting blood sample.

A 34-year-old male patient came to our outpatient department, complaining of yellow papules on his shoulders, extensor surfaces of his upper arms, and lower extremities for more than 2 months. Two months ago, the patient discovered multiple yellow papules in the above areas without apparent causes. Some papules merged without significant discomfort, but the number of rashes gradually increased, prompting him to seek medical attention. His past medical history included acute pancreatitis and fatty liver three months ago. He denied any family history of dyslipidemias, metabolic syndrome, vascular diseases, or other skin disorders, as well as any family history of similar skin lesions. He has no history of alcohol abuse or smoking, but has been accustomed to a high-fat, high-carbohydrate diet, and lack of physical exercise. After physical examination, his vital signs were stable. He is 175 cm tall and weighs 85 kg. He has a body mass index (BMI) of 27.8 kg/m^2^, which is considered overweight. Dermatological examination revealed multiple scattered and clustered yellow papules (with a diameter of about 2–5 mm) on the shoulders, extensor aspects of the upper arms, and lower extremities with a hard texture and erythematous bases (Figures 1 and 2). Some papules had a smooth surface, and no abnormalities were observed in the oral mucosa. After admission, relevant laboratory tests were conducted, and the results showed severe abnormalities in lipid and glucose metabolism (Table 1). The upper layer of the patient’ s plasma was chylous, consistent with the appearance of whole blood in patients with hyperlipidemia (Figure 3).

**Table 1:** Main laboratory test results at admission.

Test items	Results	Reference range
**Lipid profile**		

Total cholesterol (TC)	18.49 mmol/L	< 5.2 mmol/L
Triglycerides (TG)	42.36 mmol/L	< 1.7 mmol/L
High-density lipoprotein cholesterol (HDL-C)	0.54 mmol/L	≥ 1.0 mmol/L
Low-density lipoprotein cholesterol (LDL-C)	3.79 mmol/L	< 3.4 mmol/L

**Blood glucose-related**		

Fasting blood glucose	6.73 mmol/L	3.9–6.1 mmol/L
Hemoglobin (HGB)	182	130–175
Glycated hemoglobin (HbA1c)	7.3	4–6
1-Hour postprandial glucose (GLU1h)	17.69 mmol/L	–
Fasting C-Peptide (C–P0h)	7.979 ng/ml	0.3–3.73 ng/ml
Fasting insulin	41.98 uIU/ml	4.03–23.46 uIU/ml
Glucose (GLU)	6.73 mmol/L	3.9–6.1 mmol/L

**Urine-related**		

Urinary protein content (pro)	0.278 g/L	0.01–0.14 g/L
24-Hour urinary total protein (24 h-UTP)	0.389 g/24 h	0.02–0.15 g/24 h
Urinary microalbumin (MALB)	114 mg/L	2–20 mg/L
Urinary microalbumin/creatinine ratio (ACR)	156.72 mg/g creatinine	0–30 mg/g creatinine

**Inflammatory markers**		

Erythrocyte sedimentation rate (ESR)	68 mm/h	0–15 mm/h

**Informed consent:** Informed consent has been obtained from all individuals included in this study.

**Ethical approval:** The research related to human use has been complied with all the relevant national regulations, institutional policies and in accordance with the tenets of the Helsinki Declaration, and has been approved by the Medical Ethics Committee of Xingtai People’s Hospital.

### 2.2 Histopathological examination

**Figure 4:**
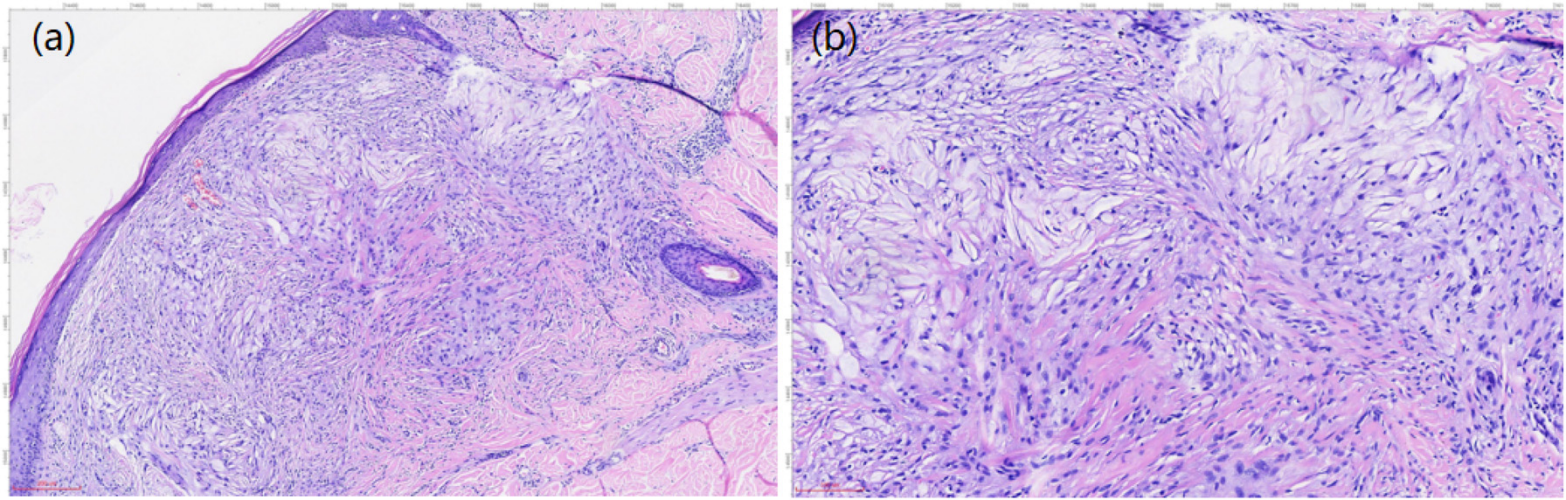
Histological examination revealed that lipid-laden macrophages aggregated into compact masses in the dermis, and the cytoplasm of these macrophages was foamy and vacuolated ((a) H&E, × 100. (b) H&E, × 200).

**Figure 5:**
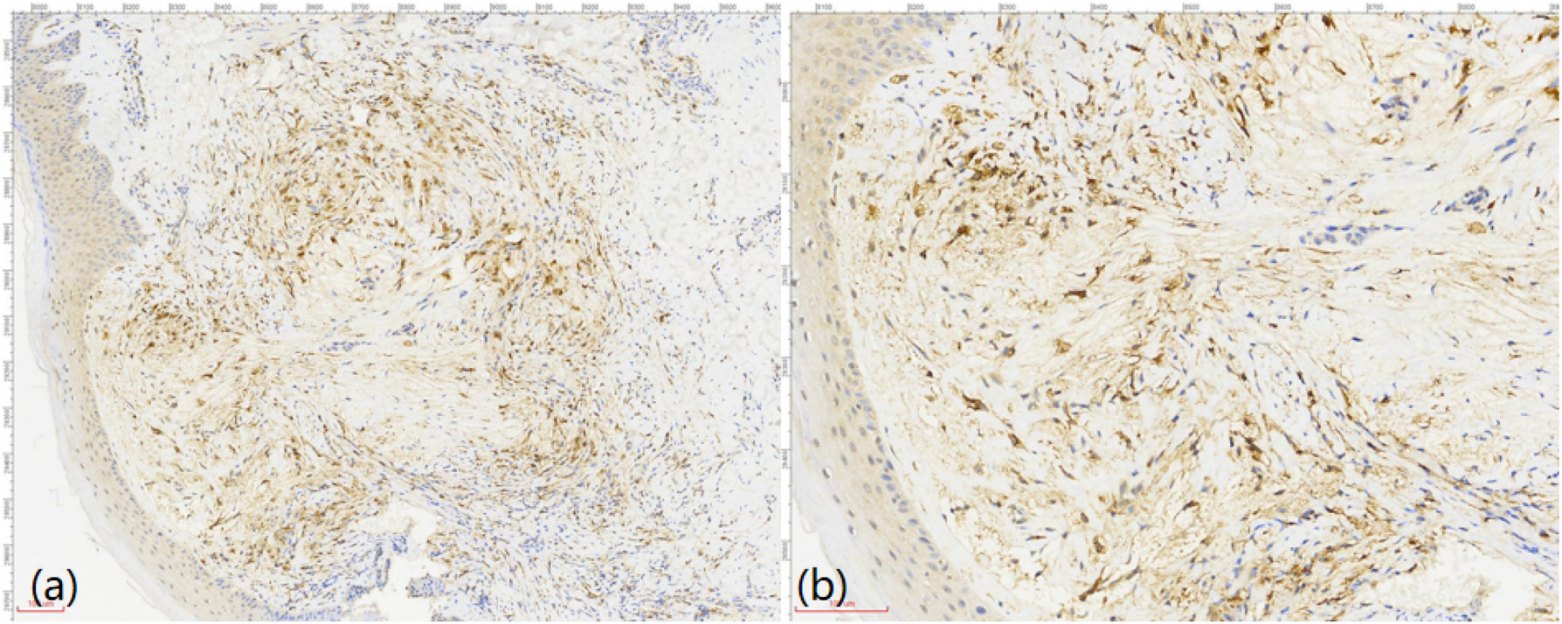
The histiocytes are positive for CD68. ((a) immunoperoxidase staining, × 100. (b) immunoperoxidase staining, × 200).

To confirm the diagnosis, a skin biopsy was performed on a lesion from the patient’s lower extremity. The specimen consisted of skin-containing soft tissue measuring 1 × 0.8 × 0.5 cm, with an epidermal area of 1 × 0.5 cm. A grayish-yellow elevation (0.4 × 0.3 × 0.1 cm) was observed on the cutaneous surface. Pathological results showed that there were a large number of foam cells with mild staining in the superficial dermis (Figure 4). Immunohistochemical staining indicated that these cells were positive for CD68 (Figure 5).

### 2.3 Diagnosis

Based on the patient’s typical cutaneous manifestations, significantly high triglyceride levels, and histopathological features, the final diagnosis is as follows:

(1) Eruptive Xanthomas
(2) Severe hypertriglyceridemia
(3) Type 2 diabetes mellitus (untreated)
(4) Overweight

## 3 Treatment and follow-up

The patient was promptly admitted to the internal medicine department for systematic treatment. The main treatment goal was to rapidly and safely reduce triglyceride levels to prevent severe complications (such as pancreatitis and coronary artery disease) and address untreated diabetes mellitus [5].

### 3.1 Treatment plan

(1) Dietary and lifestyle intervention: Start a strict low-fat diet (fat <15 % of total calories) and a low-carbohydrate diet immediately. Intake of refined sugars and alcohol was completely prohibited, and moderate aerobic exercise was recommended [5], 7], 8].
(2) Medication:
  – Lipid-lowering therapy: Given the significant increase in the patient’ triglyceride levels, we prioritized lipid-lowering therapy as the main intervention. The patient took oral fenofibrate 100 mg once a day and subcutaneous injection of tafolecimab every two weeks. Fibrates and statins are both recognized as first-line agents for the clinical management of severe hypertriglyceridemia [5], 9], 10].
  – Anti-diabetic therapy: The oral hypoglycemic drug empagliflozin (10 mg/day) was prescribed to control blood glucose. Improving blood glucose control also helps reduce triglyceride levels [5].
  – Pancreatitis treatment: Omeprazole was administered intravenously and octreotide was injected subcutaneously to inhibit gastric acid, protect the stomach and suppress pancreatic enzyme activity.

### 3.2 Follow-up results

After one week of intensive treatment, the patient’s fasting blood glucose was controlled in the normal range, and the triglyceride level rapidly decreased to 14.1 mmol/L and cholesterol was 8.75 mmol/L. After discharge, the patient continued to adhere to dietary control, exercise, and medication. At 1-month follow-up: Triglycerides decreased to 2.7 mmol/L and cholesterol was 5.8 mmol/L. The color of the skin lesions became lighter, and some papules began to flatten.

At the 3-month follow-up: Triglycerides remained stable at round 2.0 mmol/L, and cholesterol was 5.3 mmol/L. Most skin lesions had resolved, leaving only a few light brown pigmented spots. Considering the long-term risk of atherosclerotic cardiovascular disease, atorvastatin was added to the treatment regimen to further control low-density lipoprotein cholesterol (LDL-C) levels [5].

## 4 Literature review and discussion

This case has typical manifestations of eruptive xanthomas, and its diagnostic and therapeutic process emphasizes the importance of managing potential systemic diseases associated with this cutaneous marker. Based on the latest literature, we will delve into the relevant aspects of explosive xanthoma below.

### 4.1 Pathophysiology

The formation of eruptive xanthomas is a direct consequence of severe lipid metabolism imbalance. The core mechanism involves plasma triglyceride-rich lipoproteins (TRLs), mainly chylomicrons and very-low-density lipoproteins (VLDL), exceeding the normal clearance capacity of the human body [2]. This clearance impairment may be caused by a deficiency lipoprotein lipase (LPL) activity or a deficiency in its cofactor ApoC-II (primary causes), or more commonly, as a secondary to other disease states [2].

In this case, uncontrolled type 2 diabetes mellitus is a key secondary factor. Hyperglycemia and insulin resistance exacerbate hypertriglyceridemia through multiple pathways: on the one hand, insulin resistance increases the synthesis of hepatic VLDL; on the other hand, as insulin is a critical regulator of LPL activity, insulin resistance or deficiency can reduce LPL activity, thereby impairing TRL clearance [11]. When plasma TRLs is excessively elevated, these lipoproteins extravasate from capillaries into dermal tissues. Subsequently, dermal macrophages actively phagocytose these lipid particles and transform into “foam cells” filled with lipid droplets. The accumulation of these foam cells clinically present as yellow papules, namely eruptive xanthomas.

### 4.2 Clinical features and diagnosis

The diagnosis of eruptive xanthomas mainly relies on their distinctive clinical manifestations and laboratory findings.

Clinical features: A hallmark of the cutaneous lesions is their “eruptive” nature, appearing rapidly over weeks to months. They appear as yellow or orange-yellow papules (with a diameter of 1–5 mm), usually with erythematous bases. These lesions tend to occur on extensor surfaces (such as elbows and knees) and pressure-prone areas (such as buttocks and back). There may or may not be truritus or tenderness, and some patients may exhibit an isomorphic response [1].

Laboratory diagnosis and triglyceride thresholds: The gold standard for diagnosis is lipid testing. Nearly all patients with eruptive xanthomas will experience severe hypertriglyceridemia. Although there is no universally standardized diagnostic “threshold”, recent case series and research reports provide valuable reference points.

Reported triglyceride levels are typically extremely elevated such as 5,742 mg/dL and 8,869 mg/dL [7], 12], 13].

It is widely believed that the risk of eruptive xanthomas increases significantly when triglyceride levels exceed 1,000 mg/dL (11.3 mmol/L), and levels exceeding 3,500 mg/dL (approximately 39.5 mmol/L) are highly prevalent in related cases. Therefore, any patient suspected of having eruptive xanthomas should undergo immediate fasting lipid profile testing.

Differential diagnosis: Eruptive xanthomas must be distinguished from histiocytosis, juvenile xanthogranuloma, multiple milia, and molluscum contagiosum [3], 4], 14]. However, combined with typical abnormal lipid profiles, the diagnosis is usually simple.

Diagnosis of eruptive xanthomas mainly relies on a “triad” of clinical manifestations, laboratory findings, and histopathological examinations: (1) acute yellow-red papules mainly occur in friction-prone areas; (2) serum triglycerides significantly increase, usually > 1,000 mg/dL; and (3) histopathological evidence of dermal foam cells with mild lymphocytic infiltration [15]. Lipid testing is essential to distinguish between primary (such as familial LPL deficiency) and secondary (such as diabetes mellitus, alcoholic liver disease) eruptive xanthomas [16].

In different diagnosis, tuberous xanthomas (TX) are the main entities to distinguish from eruptive xanthomas. Eruptive xanthomas are typically associated with severe hypertriglyceridemia (Fredrickson hyperlipoproteinemia types I, IV, V), presenting as acute and small inflammatory skin lesions. They are mainly distributed on the surface of the extensor muscles in the buttocks and limbs, and show obvious inflammatory changes in histopathology. In contrast, tuberous xanthomas are often associated with hypercholesterolemia (type IIa) or mixed hyperlipidemia (type III), characterized by larger and non-inflammatory lesions that develop slowly. These lesions tend to occur on the extensor surfaces of joints and pressure-bearing areas, and histopathological results show mild inflammatory changes [17].

Patients with mixed hyperlipidemia may show an overlapping “tuberous-eruptive xanthoma” phenotype. In such cases, clinical evaluation only based on difference is challenging and requires a comprehensive assessment that combines lipid profiles and detailed pathological results [18].

### 4.3 Management strategies

The key to managing eruptive xanthomas lies not in treating the skin lesions, but in actively addressing their underlying cause: hypertriglyceridemia [3], 8]. Once lipid levels are effectively controlled, the skin lesions typically resolve on their own within weeks to months. Therefore, local invasive interventions (such as laser therapy or surgical excision) for the lesions are often unnecessary [3].

The management strategy is a comprehensive, multi-level approach:

(1) Main goal: For patients with triglycerides > 500 mg/dL (5.6 mmol/L), the main therapeutic objective is to prevent acute pancreatitis. Therefore, aggressive measures are required to lower triglyceride levels below this threshold [5];
(2) Lifestyle interventions: These form the foundation of all management strategies:
  – Diet: A low-fat diet < 10–15 % of total calories), strictly limiting the intake of simple carbohydrates and fructose;
  – Weight management: Weight loss is crucial for improving insulin resistance and reducing triglyceride levels;
  – Physical activity: Regular aerobic exercises;
  – Alcohol abstinence: Alcohol is a common trigger for hypertriglyceridemia [5], 7], 8].
(3) Pharmacological treatment:
  – Fibrates: Agents such as fenofibrate and gemfibrozil are first-line therapies for severe hypertriglyceridemia, which can reduce triglyceride levels by 30–50 % [5], 9], 10];
  – Omega-3 fatty acids: High-dose prescription formulations (such as icosapent ethyl) can be used as effective alternative or adjunctive treatments [3], 5], 10];
  – Niacin: This agent can effectively reduce triglycerides and raises HDL-C, but its widespread use is limited by side effects (such as flushing) [3], 10];
  – Statins: Although statins are mainly used to reduce LDL-C, they can also be used to lower triglycerides. After triglyceride levels (<500 mg/dL) are initially controlled, statins are usually added to reduce the risk of long-term atherosclerotic cardiovascular disease (ASCVD) [5];
(4) Management of emergency situations: For patients with extremely high triglyceride levels (>2000 mg/dL) or early signs of pancreatitis, more aggressive interventions (such as intravenous insulin, heparin infusion, or even plasmapheresis or triglyceride apheresis) may be needed to rapidly clear chylomicrons from plasma [5], 10], 19];
(5) Genetic testing for hypertriglyceridemia: For patients with severe hypertriglyceridemia, it is recommended to conduct genetic testing for monogenic or polygenic variants to clarify etiological subtypes, especially when secondary causes are insufficient to explain the severity of lipid abnormalities. Genetic confirmation can guide targeted treatment selection and familial risk assessment.

## 5 Conclusions

Eruptive xanthomas (EX) are a rare cutaneous manifestation of sudden appearance of yellow papules on the skin, which are also a direct external sign of severe hypertriglyceridemia. This study reports a typical case of eruptive xanthomas secondary to severe hypertriglyceridemia in a patient with uncontrolled type 2 diabetes mellitus. In addition, the patient had a history of acute pancreatitis one month before the appearance of xanthomas.

The 34-year-old male patient had yellow papules on his shoulders, extensor aspects of the upper arms, and lower extremities. Laboratory tests showed high levels of triglycerides, cholesterol, blood glucose, and acute inflammatory markers. Histopathological examination revealed foam cells in the dermis. After intervention with a low-fat and low-carbohydrate diet combined with antidiabetic, lipid-lowering, and hepatoprotective therapies, the patient’s skin lesions partially resolved within two months, and the laboratory indicators improved concomitantly. This confirms the efficacy of the treatment regimen for potential metabolic disorders. According to the analysis of this case and literature review, the diagnosis of eruptive xanthomas depends on typical clinical manifestations, blood lipid tests, and histopathological results. The core of treatment is to systematically manage potential dyslipidemia and related predisposing factors (such as diabetes mellitus).

Eruptive xanthomas and acute pancreatitis are both clinical manifestations induced by severe hypertriglyceridemia. For dermatologists, the appearance of eruptive xanthomas should be used as a trigger to screen for dyslipidemia, thereby effectively preventing life-threatening severe complications.
